# Factors Associated with Decision to Hospitalize Emergency Department Patients with Skin and Soft Tissue Infection

**DOI:** 10.5811/westjem.2014.11.24133

**Published:** 2014-12-10

**Authors:** David A. Talan, Bisan A. Salhi, Gregory J. Moran, William R. Mower, Yu-Hsiang Hsieh, Anusha Krishnadasan, Richard E. Rothman

**Affiliations:** * Olive View-UCLA Medical Center, Department of Emergency Medicine, Sylmar, California; † Emory University, Department of Emergency Medicine, Atlanta, Georgia; ‡ Ronald Reagan Medical Center, University of California at Los Angeles, Department of Emergency Medicine, Los Angeles, California; § Johns Hopkins Medical Center, Department of Emergency Medicine, Baltimore, Maryland

## Abstract

**Introduction:**

Emergency department (ED) hospitalizations for skin and soft tissue infection (SSTI) have increased, while concern for costs has grown and outpatient parenteral antibiotic options have expanded. To identify opportunities to reduce admissions, we explored factors that influence the decision to hospitalize an ED patient with a SSTI.

**Methods:**

We conducted a prospective study of adults presenting to 12 U.S. EDs with a SSTI in which physicians were surveyed as to reason(s) for admission, and clinical characteristics were correlated with disposition. We employed chi-square binary recursive partitioning to assess independent predictors of admission. Serious adverse events were recorded.

**Results:**

Among 619 patients, median age was 38.7 years. The median duration of symptoms was 4.0 days, 96 (15.5%) had a history of fever, and 46 (7.5%) had failed treatment. Median maximal length of erythema was 4.0cm (IQR, 2.0–7.0). Upon presentation, 39 (6.3%) had temperature >38°C, 81 (13.1%) tachycardia, 35 (5.7%), tachypnea, and 5 (0.8%) hypotension; at the time of the ED disposition decision, these findings were present in 9 (1.5%), 11 (1.8%), 7 (1.1%), and 3 (0.5%) patients, respectively. Ninety-four patients (15.2%) were admitted, 3 (0.5%) to the intensive care unit (ICU). Common reasons for admission were need for intravenous antibiotics in 80 (85.1%; the only reason in 41.5%), surgery in 23 (24.5%), and underlying disease in 11 (11.7%). Hospitalization was significantly associated with the following factors in decreasing order of importance: history of fever (present in 43.6% of those admitted, and 10.5% discharged; maximal length of erythema >10cm (43.6%, 11.3%); history of failed treatment (16.1%, 6.0%); any co-morbidity (61.7%, 27.2%); and age >65 years (5.4%, 1.3%). Two patients required amputation and none had ICU transfer or died.

**Conclusion:**

ED SSTI patients with fever, larger lesions, and co-morbidities tend to be hospitalized, almost all to non-critical areas and rarely do they suffer serious complications. The most common reason for admission is administration of intravenous antibiotics, which is frequently the only reason for hospitalization. With the increasing outpatient intravenous antibiotic therapy options, these results suggest that many hospitalized patients with SSTI could be managed safely and effectively as outpatients.

## INTRODUCTION

Between 1993 and 2005, annual U.S. emergency department (ED) visits for skin and soft tissue infections (SSTI) increased from 1.2 to 3.4 million,^1^ coinciding with the emergence of community-associated methicillin-resistant *Staphylococcus aureus* (MRSA).^2^ Hospitalizations for SSTI increased 29% between 2000 and 2004, whereas hospitalizations for community-acquired pneumonia (CAP) remained unchanged or decreased.^3^,^4^ Unlike CAP, guidelines for ED disposition for patients with SSTI based on validated risk stratification models do not exist.

Hospitalization is necessary for care of complicated wounds and severe sepsis, and for monitoring for acute deterioration. However, unnecessary hospitalization is expensive and associated with adverse events.^5^,^6^ Recently, the availability of outpatient parenteral antibiotic treatment strategies have expanded, which may allow alternatives to hospital admission in some cases. These strategies include use of peripherally inserted central catheters (PICC),^7^ maintenance of standard peripheral catheters with next day follow up,^8^ and administration of single-dose and weekly administered parenteral antimicrobials recently approved by the U.S. Food and Drug Administration (FDA).^9^,^10^ To identify opportunities to reduce avoidable hospitalizations for SSTI, the reasons for physician disposition decisions first need to be understood.

The primary goal of the study was to identify factors that influence the physician decision to hospitalize a patient with a SSTI. Therefore, we conducted a prospective study of adults presenting to 12 U.S. EDs in which their treating physicians were surveyed regarding reason(s) for admission, and patient clinical characteristics were correlated with hospitalization. As a secondary goal, we determined the frequency of serious sequelae.

## METHODS

### Study design and setting

We conducted a prospective study of adult patients presenting to the ED with a SSTI at 12 U.S. sites comprising *EMERGE*ncy ID NET in August of 2008.^11^,^12^

### Selection of Participants

We included patients who were ≥18 years of age, had a SSTI with symptoms present <1 week, and had purulent material available for culture. Consecutive patients were attempted to be enrolled, and an audit was conducted to compare characteristics of missed and enrolled eligible patients.^12^ Each site’s local institutional review committee approved the study. Informed consent was obtained in writing at six sites and verbally at six sites. The present study was conducted along with an analysis of SSTI bacteriology among the same study population, which was the reason that culturable material was required; the bacteriological analysis has been published.^12^

### Methods and Measurements

At the time of ED care, treating physicians completed a structured form and collected the following data (Table 1): demographics; infection duration, location, type, and mechanism; co-morbidities; symptoms; prior treatment failure for the same infection; infection-related inability to perform activities of daily living; vital sign abnormalities at triage and at the time of disposition decision; laboratory tests; presence of severe edema, lymphangitis, or extreme tenderness; maximal length and width of erythema and, if an abscess was present, estimated abscess dimension and maximal abscess depth; imaging studies; and disposition. ED providers selected the reason(s) for hospital admission from a structured list (Table 2). Abscess dimension was assessed by measurement from the apparent border on skin examination, and depth by inspection following incision and drainage. Area of erythema was estimated by the product of maximal length and width. Subjective patient and provider ratings were used to grade symptom and finding severity. We categorized a laboratory result as abnormal if it was outside the hospital’s normal range.

### Outcomes

Follow-up information collected included in-hospital duration, death, intensive care unit (ICU) admission, amputation, and/or operating room debridement/drainage.

### Analysis

We divided the enrolled cohort into admitted and discharged patients. We calculated frequencies, percentages, and relative risk for categorical outcomes, and median and interquartile ranges for continuous outcomes. Thresholds for erythema length and area were chosen based on guideline standards.^13^,^14^ We used Statpages 2-way Contingency Table Analysis to calculate relative risk and 95% confidence intervals for categorical variables.^15^ We employed chi-square binary recursive partitioning to assess for independent predictors of admission.^16^

## RESULTS

### Characteristics of study subjects

Of 619 enrolled patients, 94 (15.2%) were hospitalized; 80 (12.9%) admitted to a ward, 3 (0.5%) to the ICU, and 11 (1.8%) to an observation unit. The number of patients and admission rates varied by site from 13 to 104 and 3% to 63%, respectively. Patients’ characteristics are summarized in Table 1. Median age was 38.7 years (interquartile range [IQR], 28.0–47.6) and 57.5% were male. A comorbidity was present in 201 (32.5%) patients, with diabetes 75 (12.1%) being most common; 46 (7.5%) had prior treatment failure.

Upon presentation, 39 (6.3%) had temperature ≥38°C, 81 (13.1%) tachycardia, 35 (5.7 %) tachypnea, and 5 (0.8%) hypotension; at the time of the ED disposition decision, these findings were present in 9 (1.5%), 11 (1.8%), 7 (1.1%), and 3 (0.5%) patients, respectively.

Most infections were on the extremities. Median length of erythema was 4.0cm (IQR, 2.0–7.0). Of 579 patients (93.5%) with an abscess, 28 (4.8%) were thought to involve fascia, muscle, bone, or joint.

Laboratory tests were performed in approximately one-third of patients, including blood glucose in 177 (28.6%), white blood cell count (WBC) in 130 (21.0%), creatinine in 122 (19.7%), bicarbonate in 91 (14.7%), lactate in 16 (2.6%), and creatine phosphokinase (CPK) in 13 (2.1%). Abnormal glucose results were present in 70 (11.3%; 5 [0.81%] had glucose >500mg/dl), WBC in 72 (11.6%, 20 [3.2%] had a WBC count >15,000/mm^3^), creatinine in 15 (2.4%), bicarbonate (low) in 8 (1.3%), lactate in none, and CPK in 4 (0.65%). Imaging was performed in approximately 20% of patients; 8 (1.3%) had evidence of osteomyelitis and 3 (0.5%) had soft tissue air/gas.

### Main results

Univariate associations with hospital admission are listed in Table 1. Based on binary recursive partitioning, hospitalization was significantly associated with the following independent factors in decreasing order of importance: history of fever (present in 43.6% of those admitted, and 10.5% discharged); maximal length of erythema ≥10cm (43.6%, 11.3%); history of failed treatment (16.1%, 6.0%); any co-morbidity (61.7%, 27.2%); and age ≥65 years (5.4%, 1.3%). At least one of these characteristics was present in 89 of the 94 admitted patients, while all were absent in 291 of 525 discharged patients.

Reasons cited by the treating physician to admit their patient to the hospital are summarized in Table 2. Need for intravenous antibiotics was the most common reason for admission, cited for 80 (85.1%) patients, and was the only reason for admission in 39 (41.5%). The next most common reasons were need for surgical intervention in 23 (24.5%), significant underlying disease in 11 (11.7%), and complex wound care in 9 (9.6%).

Median hospital duration was four days (IQR, 2–6). Among admitted cases, debridement/drainage in the operating room, amputation, subsequent ICU admission, and/or death occurred in 20 (21.3%), 2 (2.1%), 0 (0%), and 0 (0%), respectively. One patient who underwent amputation was admitted to the floor with diabetes and a foot ulcer infection with a 30 × 15cm area of cellulitis, and initial radiograph demonstrating soft tissue gas. The second patient requiring amputation was also a diabetic who was admitted to the ICU with a large cellulitis area, 20 × 12cm, soft tissue gas on radiograph, and a WBC count of 34,000/mm^3^.

## DISCUSSION

ED visits and hospitalizations for SSTI have greatly increased over the last decade.^1^,^3^ One study estimated that there were approximately three million ED visits for SSTI and 500,000 associated hospitalizations annually based on data from 2008 through 2010.^15^ The estimated mean cost of an SSTI hospitalization in the U.S. is $8,023 with a 4.9 day length of stay, and hospitalization is also associated with various risks.^5^,^6^ In this study, we sought to identify factors that may affect the decision to hospitalize an ED patient with a SSTI. We also determined the rate of major procedures and serious complications that may justify hospitalization. Among 619 patients, 15.2% were admitted, and only 0.5% were admitted to the ICU. Even among admitted patients, vital sign abnormalities at the time of ED discharge were rare. For the first time that we are aware, we surveyed treating physicians at the time of their care decisions as to their reasons for hospitalizing a patient with a SSTI. We found that the most common reason for hospital admission by far was perceived need for intravenous antibiotics, cited for 85.1% of patients. Administration of intravenous antibiotics was the sole reason for hospitalization for 41.5% of patients. A patient’s inability to take oral medication was rarely cited as a reason for admission. Anticipated need for major surgery or wound management was indicated for about one-quarter. While EPs tended to hospitalize patients who had fever, larger lesions, failed treatment, co-morbidities and advanced age, of 94 admitted patients, none had subsequent ICU transfer or died, and only two had amputations, both of whom had soft tissue gas on their initial radiographs. In light of expanded options for outpatient parenteral antibiotic administration, it appears that a substantial proportion of ED patients hospitalized for SSTI could instead be safely and effectively managed as outpatients.

Unlike CAP, robust outcome data that would inform admission decisions have never been reported for patients with SSTI. This is not surprising since, as we observed, serious adverse events are rare in this infection compared to CAP, with CAP hospitalization rates over 50% and inpatient mortality estimated at 8–14%.^4^,^18^–^21^ In contrast, and consistent with our observations, an analysis of over eight million adults presenting with SSTI using U.S. Healthcare Cost and Utilization Project Nationwide Emergency Department Sample (HCUP NEDS) data from 2008 to 2010 found a hospitalization rate of 17% and an inpatient mortality rate of only 0.5%.^17^

To the best of our knowledge, this is also the first ED-based study of a large group of patients with SSTI in which a broad range of patient characteristics was collected prospectively and examined for their association with patient disposition. We found factors independently associated with hospitalization were history of fever, maximal wound dimension ≥10cm, history of failed treatment, presence of any co-morbidity, and age ≥65 years. One retrospective ED investigation by Sabbaj et al.^20^ described 846 patients and also found that fever was associated with hospitalization.

Hospital admission is neither required to administer parenteral antibiotics nor to achieve good patient outcomes, even among patients with fever, large areas of cellulitis, and co-morbidities. Newly FDA-approved parenteral lipoglycopetides, dalbavancin and oritavancin, have exceptionally long half-lives that allow either a single dose or two doses, one week apart, which could be initiated in the ED prior to discharge.^9^,^10^ Two randomized, double-blind, double-dummy, trials comparing dalbavancin, two injections one week apart to intravenous vancomycin, at least three days followed by oral linezolid, found similar response rates among 1,315 SSTI patients, the majority of whom were hospitalized.^10^ Subjects had frequent fever (84% had temperature ≥38.0°C) and very large areas of erythema (median, 313–367cm^2^, about the size of a standard tablet portable computer). Approximately 13% had diabetes. The median area of erythema among subjects in this clinical trial was substantially larger than that of patients who were hospitalized in our study (i.e., estimated median area ~48cm^2^). Among all clinical trial subjects, there was one case of necrotizing fasciitis and no septic deaths. Approximately 25% of subjects were treated entirely as outpatients.

Other alternatives to inpatient administration of intravenous antibiotics include peripherally inserted central catheters (PICC) for outpatient parenteral antibiotic treatment (OPAT).^7^ One innovative approach is to administer a once-a-day parenteral agent prior to ED discharge, leaving the standard peripheral intravenous catheter for next day follow-up and repeat dosing in the ED or infectious diseases clinic.^8^,^23^ Oral antibiotics, with good compliance, may also be an alternative in some cases. In a retrospective, propensity score-matched case-control study of adults with complicated SSTI, of whom about 20%–30% had diabetes and/or peripheral vascular disease, oral linezolid actually was associated with a greater chance of clinical cure than intravenous vancomycin.^24^

Some risk-stratification models based on hospital SSTI populations exist that might guide ED disposition decisions but are limited by small size, selected patient populations and low rates of serious adverse events. Figtree et al.^25^ reported outcomes among 395 adults admitted to a referral hospital; 2.5% of patients died, 5.1% had multi-organ failure, and 0.8% had amputation. A predictive model for adverse outcomes was derived consisting of a weighted score based on altered mentation, heart failure, wound discharge, hypoalbuminemia, and neutrophilia/neutropenia. Carralta et al.^26^ analyzed 332 adults hospitalized with SSTI. Thirty-day mortality was 5% and factors associated with death were male gender, comorbidities, heart failure, obesity, hypoalbuminemia, renal insufficiency, shock, and *Pseudomonas* cellulitis. The laboratory risk indicator for necrotizing fasciitis is a weighted risk-stratification scoring system based on abnormalities of serum sodium, glucose, creatinine, hemoglobin, WBC, and C-reactive protein derived among hospitalized SSTI patients to diagnose necrotizing fasciitis.^27^ Unvalidated expert-based disposition guidelines have been proposed, 28–33 one combining a graded scale of vital sign abnormalities and altered mentation, and presence of sepsis and/or significant co-morbidities.^31^

## LIMITATIONS

In this study, physician survey responses and clinical correlates with hospitalization may not reflect the actual reason(s) an emergency physician decided that a patient required hospital care. Admission decisions may be influenced by factors not analyzed, such as by a patient’s primary care physician, whose reasoning may not have been reflected by the emergency provider. However, other than asking the treating physician at the time of their care, and examining clinical findings present at the time for their association with admission, we are unaware of a better way to assess provider justification for hospitalization. We did not collect outcome data on discharged patients, although the risk of adverse outcomes would be expected to be substantially less than among admitted patients. Study sites were urban, university-affiliated hospitals that may not reflect practices in other settings. Admission rates varied greatly among sites, from 3% to 63%. This likely reflects sampling issues and case-mix, with a few sites enrolling a small number of patients. For example, the frequency of fever history among subjects at sites with the highest and lowest hospitalization rates was 47% and 9%, respectively. However, the rate of hospitalization predictors among admitted patients by site was similar. Variation in admission rates by site also suggests variation in practice patterns, perhaps related to availability outpatient care services and differences in payer models, and supports a range of acceptable approaches to SSTI management. Because another study purpose was bacteriological analysis, the study population was patients with purulent SSTI, mostly patients with abscesses and a minority of patients with cellulitis or wound infection, and some drainage,^12^ who may be different than other SSTI patients. While our sample does not include patients with cellulitis without drainage, it would be expected that these patients would be more likely to be hospitalized for parenteral antibiotics and not for surgery or wound care than those with purulent drainage. Enrolled patients may be different from all eligible patients, although case finding audits have found these groups to be similar.^2^,^12^ While our study population of ED patients with SSTI may therefore not reflect all such patients, importantly our admission and hospital mortality rates were similar to those of two large U.S. databases for patients with SSTI diagnoses.^5^,^17^ Some univariate associations with hospitalization may have been artifactual, and therefore, we conducted a multivariate analysis to identify independent predictors. However, these independent associations may be an over-simplification of the factors that are the bases for provider admission decisions.

## CONCLUSION

ED patients with SSTI with fever, larger lesions, failed prior treatment, co-morbidities and advanced age tend to be hospitalized. The most common reason given by treating clinicians for admission is administration of intravenous antibiotics, which was frequently the only reason for hospitalization. Almost all these patients are admitted to non-critical care areas and rarely do they suffer serious adverse events. In light of the increasing availability of outpatient intravenous antibiotic therapy options, these results suggest that many hospitalized patients with SSTI could be managed safely and effectively as outpatients. Since this was not a clinical trial, we can only surmise based on other existing evidence that outcomes of low- and moderate-risk patients admitted only for intravenous antibiotics would be as good as if these patients had been discharged and treated with various outpatient parenteral antibiotic strategies or even oral antibiotics. It would be ideal to collect sufficient outcome data to develop a validated risk-stratification model, along the lines of the Pneumonia Severity Index.^34^ Implementation of these tools has been demonstrated to reduce CAP hospital admission rates.^4^,^35^ Because of the relatively low rate of serious complications associated with SSTI, case series and clinical trials may be more appropriate than prospective cohort studies to address alternative management options to hospitalization and intravenous antibiotics for stable patients with SSTI.

## Figures and Tables

**Table 1:** Demographic and clinical characteristics of 619 U. S. emergency department patients with a skin and soft tissue infection, by admission or discharge.

Characteristics	All patients (n=619*)	Admitted (n=94)	Discharged (n=525)	Relative risk [95% CI]
Age (years; median [IQR])	38.7 [28.0, 47.6]	44.0 [36.2, 52.0]	37.2 [26.7, 47.0]	N/A
≥65 years - n/total (%)	12/66 (2.0)	5/93 (5.4)	7/523 (1.3)	2.86 [1.11 – 5.08]†
Gender - n (%)				
Male	356 (57.5)	59 (62.8)	297 (56.6)	1.25 [0.83 – 1.88]
Female	263 (42.5)	35 (37.2)	228 (43.4)	0.80 [0.53 – 1.20]
Race - n (%)				
White	283 (45.7)	58 (61.7)	225 (42.9)	1.91 [1.28 – 2.88]†
Black	319 (51.5)	35 (37.2)	283 (53.9)	0.56 [0.37 – 0.84]†
Other	17 (2.7)	1 (1.1)	16 (3.0)	0.38 [0.06 – 2.57]
Ethnicity - n (%)				
Hispanic	138 (22.3)	17 (18.1)	121 (23.0)	0.77 [0.45 – 1.27]
Co-morbidity - n (%)				
Any	201 (32.5)	58 (61.7)	143 (27.2)	3.35 [2.25 – 5.00]†
Prior MRSA infection	56 (9.0)	14 (14.9)	42 (8.0)	1.76 [1.00 – 2.89]†
Diabetes	75 (12.1)	23 (24.5)	52 (9.9)	2.35 [1.50 – 3.53]†
Peripheral vascular disease	6 (1.0)	3 (3.2)	3 (0.6)	3.37 [0.92 – 5.90]
Eczema or other chronic skin condition	9 (1.5)	4 (4.3)	5 (1.0)	3.01 [1.02 – 5.41]†
Chronic ulcer in area of infection	1 (0.2)	1 (1.1)	0 (0.0)	6.65 [0.36 – 6.65]
Chronic edema	6 (1.0)	4 (4.3)	2 (0.4)	4.54 [1.61 – 6.52]†
Chronic renal failure	4 (0.6)	0 (0.0)	4 (0.8)	0.0 [0.0 – 4.04]
Chronic liver failure	4 (0.6)	1 (1.1)	3 (0.6)	1.51 [0.08 – 4.80]
COPD	8 (1.3)	3 (3.2)	5 (1.0)	2.52 [0.68 – 5.13]
CHF	3 (0.5)	1 (1.1)	2 (0.4)	2.21 [0.44 – 11.1]
Pregnancy	3 (0.5)	0 (0.0)	3 (0.6)	0.0 [0.0 – 4.61]
HIV	31 (5.0)	9 (9.6)	22 (4.2)	2.01 [1.00 – 3.51]†
Cancer	6 (1.0)	1 (1.1)	5 (1.0)	1.10 [0.06 – 4.30]
Bedridden/paralysis	1 (0.2)	0 (0.0)	1 (0.2)	0.0 [0.0 – 6.28]
Infection type - n (%)				
Abscess	527 (85.1)	63 (67.0)	464 (88.4)	0.35 [0.25 – 0.51]†
Infected wound	55 (8.9)	14 (14.9)	41 (7.8)	1.79 [1.09 – 2.95]†
Cellulitis	37 (6.0)	17 (18.1)	20 (3.8)	3.47 [2.31 – 5.22]†
Infection mechanism - n (%)				
Recent wound or other break in the skin	114 (18.4)	20 (21.3)	94 (17.9)	1.20 [0.73 – 1.90]
Chronic wound	3 (0.5)	3 (3.2)	0 (0.0)	6.77 [2.06 – 6.77]†
IVDU	25 (4.0)	13 (13.8)	12 (2.3)	3.81 [2.23 – 5.54]†
Insect/spider bite	106 (17.1)	9 (9.6)	97 (18.5)	0.51 [0.25 – 1.00]
Animal/human bite	3 (0.5)	1 (1.1)	2 (0.4)	2.28 [0.12 – 5.89]
Burn infection	1 (0.2)	1 (1.1)	0 (0.0)	6.65 [0.36 – 6.65]
Infection of surgical wound	16 (2.6)	8 (8.5)	8 (1.5)	3.51 [1.73 – 5.44]†
No apparent precipitating event	304 (49.1)	30 (31.9)	274 (52.2)	0.49 [0.32 – 0.74]†
Other	47 (7.6)	9 (9.6)	38 (7.2)	1.29 [0.63 – 2.38]
Infection location - n (%)				
Head/neck	57 (9.2)	8 (8.5)	49 (9.3)	0.92 [0.42 – 1.80]
Torso	101 (16.3)	12 (12.8)	89 (17.0)	0.75 [0.40 – 1.34]
Groin/perineum/buttock	132 (21.3)	12 (12.8)	120 (22.9)	0.54 [0.29 – 0.97]†
Upper extremity	174 (28.1)	27 (28.7)	147 (28.0)	1.03 [0.66 – 1.58]
Lower extremity	172 (27.8)	37 (39.4)	135 (25.7)	1.69 [1.13 – 2.49]†
Failed prior treatment for same infection - n (%)	46/611 (7.5)	15/93 (16.1)	31/518 (6.0)	2.36 [1.38 – 3.72]†
Total symptom duration (days; median [IQR])	4.0 [3.0, 6.0]	4.0 [3.0, 6.0]	4.0 [3.0, 6.0]	N/A
Symptoms - n (%)				
Fever	96 (15.5)	41 (43.6)	55 (10.5)	4.21 [2.92 – 5.96]†
Chills/sweats/rigors	99 (16.0)	31 (33.0)	68 (13.0)	2.59 [1.72 – 3.78]†
Severe nausea/vomiting	28 (4.5)	10 (10.6)	18 (3.4)	2.51 [1.31 – 4.14]†
Extreme pain	264 (42.6)	53 (56.4)	211 (40.2)	1.74 [1.17 – 2.59]†
Lymphangitis	31/535 (5.8)	15/84 (17.9)	16/451 (3.5)	3.53 [2.12 – 5.21]†
Marked local edema	275/576 (47.7)	68/91 (70.1)	207/485 (42.7)	3.24 [2.05 – 5.22]†
Extreme tenderness	381/594 (64.1)	73/91 (75.3)	308/503 (61.2)	2.27 [1.37 – 3.86]†
ED Vital Sign Abnormalities - n (%)				
Temperature ≥38°C	39 (6.3)	20 (21.3)	19 (3.6)	4.02 [2.60 – 5.66]†
Hypotension (SBP <90mmHg)	5 (0.8)	2 (2.1)	3 (0.6)	2.67 [0.48 – 5.66]
Tachycardia (>100/minute)	81 (13.1)	29 (30.9)	52 (9.9)	2.96 [1.97 – 4.30]†
Tachypnea (≥20/minute)	35 (5.7)	12 (12.8)	23 (4.4)	2.44 [1.35 – 3.94]†
Disposition vital sign abnormalities - n (%)				
Temperature ≥38°C	9 (1.5)	5 (5.3)	4 (0.8)	3.81 [1.52 – 5.97]†
Hypotension (SBP <90mmHg)	3 (0.5)	2 (2.1)	1 (0.2)	4.46 [1.96 – 10.1]†
Tachycardia (>100/minute)	11 (1.8)	4 (4.3)	7 (1.3)	2.46 [0.82 – 4.78]
Tachypnea (≥20/minute)	7 (1.1)	2 (2.1)	5 (1.0)	1.90 [0.34 – 4.77]
Size of wound-erythema (cm)				
Maximal length - median [IQR] n=589	4.0 [2.0, 7.0]	8.0 [4.0, 10.0]	4.0 [2.0, 6.0]	N/A
Maximal width - median [IQR] n=588	3.0 [2.0, 5.0]	6.0 [4.0, 10.0]	3.0 [2.0, 5.0]	N/A
Max dimension ≥5cm - n (%)	262/589 (44.5)	72/94 (76.6)	190/495 (38.4)	4.09 [2.57 – 6.62]†
Max dimension ≥10cm - n (%)	97/589 (16.5)	41/94 (43.6)	56/495 (11.3)	3.92 [2.72 – 5.56]†
Area ≥19.7cm^2^ - n (%)	203/588 (34.5)	64/94 (68.1)	139/494 (28.1)	4.05 [2.68 – 6.18]†
Area ≥78.5cm^2^ - n (%)	64/589 (10.9)	30/94 (31.9)	34/495 (6.9)	3.85 [2.62 – 5.39]†
Size of abscess (cm)				
Maximal length - median [IQR] n=534	2.0 [1.0, 3.5]	4.0 [2.0, 10.0]	2.0 [1.0, 4.0]	N/A
Maximal width - median [IQR] n=571	2.0 [1.0, 3.0]	3.0 [2.0, 8.0]	2.0 [1.0, 3.0]	N/A
Abscess depth - n (%)				
Limited to skin	551/579 (95.2)	68/83 (81.9)	483/496 (97.4)	0.23 [0.16 – 0.38]†
Involves deep fascia/muscle	26/579 (4.5)	15/83 (18.1)	11/496 (2.2)	4.69 [2.87 – 6.59]†
Involves bone/joint	2/579 (0.3)	0/83 (0.0)	2/496 (0.4)	0.00 [0.00 – 5.68]
Laboratory tests - n (%)‡				
WBC‡ – performed	130 (21.0)	86 (91.5)	44 (8.4)	40.4 [20.4 – 87.1]†
WBC – abnormal	72/130 (55.4)	51/86 (59.3)	21/44 (47.7)	1.17 [0.90 – 1.55]
Creatinine – performed	122 (19.7)	78 (83.0)	44 (8.4)	19.9 [12.2 – 33.4]†
Creatinine – abnormal	15 (12.3)	13/78 (16.7)	2/44 (4.5)	1.43 [0.93 – 1.65]
HCO_3_ – performed	91 (14.7)	55 (58.5)	36 (6.9)	8.18 [5.79 – 11.4]†
HCO_3_ – abnormal	8 (8.8)	7/55 (12.7)	1/36 (2.8)	1.51 [0.78 – 1.75]
Lactate – performed	16 (2.6)	11 (11.7)	5 (1.0)	5.00 [2.91 – 6.62]†
Lactate – abnormal	0 (0)	0 (0)	0 (0)	N/A
Glucose – performed	177 (28.6)	79 (84.0)	98 (18.7)	13.2 [7.74 – 23.2]†
Glucose – abnormal	70 (39.5)	35/79 (44.3)	35/98 (35.7)	1.22 [0.85 – 1.71]
CPK‡ – performed	13 (2.1)	10 (10.6)	3 (0.6)	5.55 [3.21 – 6.95]†
CPK – abnormal	4 (30.8)	3/10 (30.0)	1/3 (33.3)	0.96 [0.38 – 1.47]
Imaging performed - n (%)				
Ultrasound	32 (5.2)	8 (8.5)	24 (4.6)	1.71 [0.80 – 3.13]
X-ray	67 (10.8)	38 (40.4)	29 (5.5)	5.59 [3.95 – 7.58]†
CT	22 (3.6)	11 (11.7)	11 (2.1)	3.60 [2.00 – 5.38]†
MRI	4 (0.6)	3 (3.2)	1 (0.2)	5.07 [1.46 – 6.74]†
Any	115 (18.6)	53 (56.4)	62 (11.8)	5.67 [3.93 – 8.12]†
Imaging results - n (%)				
Abscess	45 (7.3)	15 (16.0)	30 (5.7)	2.42 [1.42 – 3.80]†
Gas/air in tissues	3 (0.5)	2 (2.1)	1 (0.2)	4.46 [0.83 – 6.65]
Osteomyelitis	8 (1.3)	7 (7.4)	1 (0.2)	6.15 [3.19 – 7.05]†
Other abnormality	22 (3.6)	14 (14.9)	8 (1.5)	4.75 [2.92 – 6.41]†
Debridement procedure in the ED - n (%)	538/615 (87.5)	66 (70.2)	472/521 (90.6)	0.34 [0.23 – 0.51]†
Culture results - n (%)				
Any bacterial growth	580 (93.7)	88 (93.6)	492 (93.7)	0.99 [0.47 – 2.43]
MRSA‡	366 (59.2)	45 (47.9)	321 (61.1)	0.64 [0.43 – 0.94]†
MSSA‡	94 (15.2)	16 (17.0)	78 (14.9)	1.15 [0.66 – 1.89]
β-hemolytic streptococci	36 (5.8)	9 (9.6)	27 (5.1)	1.72 [0.84 – 3.06]
Viridans streptococci	23 (3.7)	7 (7.4)	16 (3.0)	2.09 [0.94 – 3.80]

*COPD,* chronic obstructive pulmonary disease; *CHF,* congestive heart failure; *HIV,* human immunodeficiency virus; *IVDU,* intravenous drug users; *ED,* emergency department; *SBP,* systolic blood pressure; *WBC*, white blood cell count; *CPK,* creatine phosphokinase; *CT,* computed tomography; *MRI*, magnetic resonance imaging; *MRSA,* methicillin-resistant *S. aureus*; *MSSA*, methicillin-sensitive *S. aureus*

* Subjects with unknown or missing values for characteristics were excluded from calculations. Denominators are provided for the number of subjects with complete data.

† Statistically significant associations.

‡ For abnormal tests, the number and percent abnormal of those with test performed is shown.

**Table 2:** Physician reasons for admission among 94 emergency department patients hospitalized with a skin and soft tissue infection.

Reason for admission	Frequency (%)*
Needs intravenous antibiotics	80 (85.1)
Needs surgical intervention	23 (24.5)
Needs complex wound care	9 (9.6)
Unable to tolerate oral medications	0 (0.0)
Needs pain control	5 (5.3)
Significant underlying disease	11 (11.7)
Homeless	2 (2.1)
Unreliable for taking medications	4 (4.3)
Possible necrotizing fasciitis	2 (2.1)
Possible deep vein thrombosis	1 (1.1)
Possible osteomyelitis/septic arthritis	5 (5.3)
Possible endocarditis	2 (2.1)
Possible sepsis/bacteremia	4 (4.3)

* More than one reason could be given.
